# Survival Rate and Biological Complications of Screw‐Retained Ceramic‐Veneered Implant‐Supported Fixed Dental Prostheses: A Systematic Review and Meta‐Analysis

**DOI:** 10.1155/bmri/5326872

**Published:** 2026-06-25

**Authors:** Mojtaba Sheykhian, Javad Hayati Garjan, Ali Sharifi, Mehran Rahbar

**Affiliations:** ^1^ Department of Oral and Maxillofacial Surgery, Faculty of Dentistry, Tabriz University of Medical Sciences, Tabriz, Iran, tbzmed.ac.ir

**Keywords:** ceramics, dental implants, dental prosthesis, implant-supported, prostheses and implants

## Abstract

**Aim:**

The present systematic review and meta‐analysis were aimed at evaluating the survival rate and biological complications of screw‐retained ceramic‐veneered implant‐supported fixed dental prostheses (ISFDPs).

**Method:**

The design and implementation of the present systematic review and meta‐analysis were based on the 27‐item PRISMA 2020 checklist. A search was conducted based on keywords related to the research objective in the international databases (Cochrane, Embase, and MEDLINE [PubMed and Ovid]) until January 2025. The quality and risk of bias of the studies were assessed using the standard Newcastle–Ottawa Scale. In the present study, survival rate, success rate, and complication rate were evaluated with a fixed effects model at a 95% confidence level; Stata 17 software was used to perform meta‐analysis.

**Result:**

The survival rate of screw‐retained ceramic‐veneered ISFDPs was 97% (effect size [ES]: 0.97; 95% CI: 0.92–1.09; *I* ^2^ = 0.0*%*, *Q* = 3.41, *p* = 0.7565). The complication rate of screw‐retained ceramic‐veneered ISFDPs was 13% (ES: 0.13; 95% CI: 0.08–0.18; *I* ^2^ = 5.7*%*, *Q* = 6.36, *p* = 0.3839).

**Conclusion:**

Screw‐retained ceramic‐veneered ISFDPs had a high and acceptable success rate and survival rate.

## 1. Introduction

Implant‐supported fixed dental prostheses (ISFDPs) are widely recognized as a reliable and predictable treatment modality for the rehabilitation of partial and complete edentulism, demonstrating high long‐term survival rates [1, 2]. Recent advancements in computer‐aided design and computer‐aided manufacturing (CAD‐CAM) technologies have facilitated the expanded use of various restorative materials, including ceramics, zirconia, and titanium, in implant‐supported reconstructions [3].

Several studies have reported favorable clinical outcomes for ISFDPs supported by veneered zirconia frameworks, with high survival rates and satisfactory esthetic results [4]. Nevertheless, ceramic chipping remains one of the most frequently reported mechanical complications associated with veneered zirconia prostheses. As a result, monolithic zirconia restorations have been proposed as an alternative approach, potentially reducing the incidence of veneering ceramic fractures [5].

Previous investigations suggest that the type of prosthesis retention, screw‐retained or cement‐retained, does not significantly influence overall prosthesis survival. However, biological and technical complications related to retention type, such as peri‐implant soft tissue inflammation, screw loosening, and prosthetic fractures, remain clinically relevant and should not be overlooked [5, 6]. Most available evidence has focused on implant‐supported fixed prostheses in general, with limited attention to specific prosthetic designs and retention mechanisms. According to earlier reports, porcelain‐fused‐to‐metal (PFM) ISFDPs demonstrate survival rates of approximately 96.4% after 5 years and 93.9% after 10 years of follow‐up [7, 8]. Despite these favorable outcomes, comprehensive data regarding implant failure, prosthetic failure, and biological complications specifically associated with screw‐retained ceramic‐veneered ISFDPs are still scarce. Therefore, the present systematic review and meta‐analysis are aimed at evaluating implant and prosthetic survival rate, as well as biological complications, in screw‐retained ceramic‐veneered ISFDPs.

Hypothesis: Screw‐retained ceramic‐veneered ISFDPs demonstrate high survival and success rates with a low incidence of biological and technical complications.

## 2. Method

### 2.1. Search Strategy and Selection Criteria

An initial search was conducted in international databases up to January 2025, with full reports presented in Table 1. In order to complete the sources and avoid missing relevant articles, the references of the articles were also checked, and Google Scholar was also searched. All parts of the present study, from design to implementation, were conducted based on PRISMA 2020 with 27 items [9]. This systematic review was not registered in the PROSPERO database.

**Table 1:** Search based on relevant keywords in the international database.

Databases	Keywords
MEDLINE (via PubMed)	(((((((“Dental Prosthesis”[Mesh] OR “Dental Prosthesis, Implant‐Supported”[Mesh] OR “Dental Implants”[Mesh] OR “Dental Restoration, Temporary”[Mesh]) OR (“Dental Prosthesis/adverse effects”[Mesh] OR “Dental Prosthesis/methods”[Mesh] OR “Dental Prosthesis/statistics and numerical data”[Mesh])) OR (“Dental Prosthesis, Implant‐Supported/adverse effects”[Mesh] OR “Dental Prosthesis, Implant‐Supported/methods”[Mesh])) AND “Ceramics”[Mesh]) AND “zirconium oxide” [Supplementary Concept]) AND (“Survival”[Mesh] OR “Survival Analysis”[Mesh] OR “Survival Rate”[Mesh])) AND “complications” [Subheading]) AND “Prosthesis Failure”[Mesh]
Cochrane	“Implant‐supported fixed dental prostheses” AND “Screw‐retained ceramic‐veneered” OR “monolithic zirconia partial” AND “Prosthesis Failure” AND “Survival Rate” AND “complications”
Embase	implant‐supported fixed dental prostheses AND Screw‐retained ceramic‐veneered OR monolithic zirconia partial: ab,ti,kw Prosthesis Failure AND complications AND Survival Rate: ab,ti,kw

Only articles published in English were included in this systematic review. To accurately answer the research questions, the PICO framework was used, in which:

Population (P): patients with a dental condition of missing more than two teeth in the upper jaw, lower jaw, or both jaws.

Intervention (I): screw‐retained ceramic‐veneered ISFDPs.

Comparison (C): not applicable.

Outcome (O): survival rate, success rate, and complication rate.

Exclusion criteria: Studies with designs other than randomized clinical trials (RCTs) or cohort studies.

### 2.2. The Process of Selection and Data Collection

Two independent, blinded investigators reviewed all study data, unaware of each other′s assessments, and consensus was confirmed by a third investigator, resolving any disagreements. The data were collected based on a predesigned template by the researchers, which included the name of the first author, year of publication, study method, number of participants, gender, mean age, jaw location, types of dentures, and implant category.

### 2.3. Statistical Heterogeneity

To assess the degree of heterogeneity among the included studies, the chi‐square (*χ* ^2^) test and the *I* ^2^ index were used. The *I* ^2^ value was evaluated at four levels to interpret the severity of heterogeneity: low heterogeneity: less than or equal to 25%; medium heterogeneity: between 25% and 50%; high heterogeneity: between 50% and 75%; and very high heterogeneity: more than 75%.

### 2.4. Methodological Quality Assessment Tools

The Newcastle–Ottawa Scale (NOS) was used to assess the risk of bias in the included cohort studies [10]. In this scale, the assessment is based on three main components: selection of study groups, comparability of groups, and measurement of outcomes, and a score between 0 and 9 is assigned to each study. Based on the total score, there is a high (0–3), moderate (4–6), and low (7–9) risk of bias. Two independent, blinded investigators reviewed all study data, unaware of each other′s assessments. Inter‐rater agreement between the two reviewers was assessed, and any discrepancies were resolved through discussion with a third investigator until consensus was achieved.

### 2.5. Data Analysis

The survival, success, and biological complication rates were used as an effect size (ES) with a fixed‐effects model and inverse‐variance methods of 95% confidence intervals (CIs). A random‐effects model was used to account for moderate to high heterogeneity among the included studies in terms of study design, follow‐up duration, and prosthesis characteristics. The DerSimonian and Laird method was used for pooled estimates. A sensitivity analysis was performed to assess the robustness of the findings by excluding studies with a moderate risk of bias. Meta‐analysis was performed using Stata (as of Version 17). Statistical significance was considered less than 0.05.

## 3. Result

### 3.1. Description of Studies

**Figure 1:**
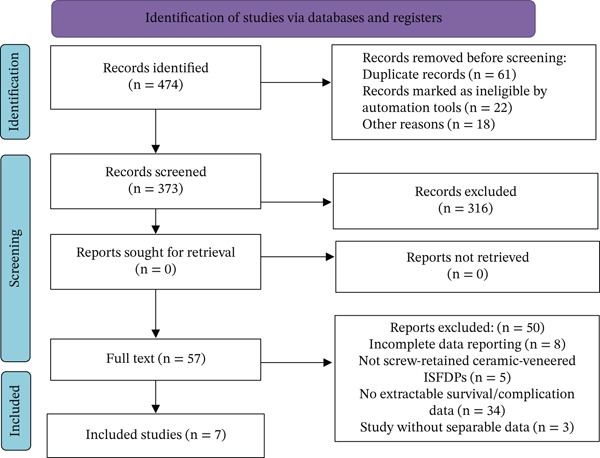
PRISMA 2020 checklist.

The initial search yielded 474 articles. After removing duplicate and irrelevant articles, 373 articles entered the title and abstract review stage. According to the inclusion and exclusion criteria, 316 articles were excluded from the study at this stage. Then, the full text of 57 articles was carefully evaluated by two independent researchers, and finally, articles that were irrelevant to the study objective, presented incomplete data, did not present a clear methodology, and articles of very low quality were excluded, and seven articles were selected for meta‐analysis (Figure 1).

### 3.2. Study Characteristics

A total of 694 patients were included in the present meta‐analysis, comprising 369 females and 325 males, with a mean age of 56.58 years. Overall, 663 ISFDPs supported by 1246 implants were analyzed. The main characteristics of the included studies are summarized in Table 2.

**Table 2:** Summary of characteristics of studies selected for the present meta‐analysis.

Study, year	Study design	No. of patients	Gender		Mean age	No. of prostheses	No. of implants	Prosthesis type	Material/veneering	Follow‐up duration	Complication type breakdown
			Female	Male							
Pontevedra et al., 2024 [11]	ReS	60	38	22	60.2	60	60	Short‐span (three‐unit FDPs)	MZ, VZ, and MC	5 years	Chipping, fracture, retention loss, and biological
Zhang et al., 2024 [2]	ReS	171	83	88	61.1	208	451	Short‐span (two to four units)	Zirconia/monolithic + veneered	5–10 years	Technical + biological
Solá‐Ruiz et al., 2022 [12]	Pro	27	14	13	60	27	27	Three to six units (partial)	Zirconia/veneered (feldspathic porcelain)	12 years	Technical + biological
Pontevedra et al., 2022 [13]	ReS	60	38	22	56	60	60	Fixed partial denture (three units)	Zirconia/monolithic + veneered	3 years	Technical + biological
De Angelis et al., 2021 [14]	ReS	25	14	11	56.9	25	25	Short‐span (Kennedy Class II)	Zirconia/monolithic + partial veneer	3 years	Screw loosening, chipping, and fracture
Diéguez‐Pereira et al., 2020 [15]	ReS	48	22	26	45.3	154	112	Three‐unit FDPs and four to five‐unit prostheses	Zirconia/monolithic + buccal veneer	NR	Mechanical + technical
Wittneben et al., 2014 [16]	ReS	303	160	143	NR	129	511	Single crowns, full‐arch reconstructions, and FDP	Metal–ceramic/all‐ceramic	10 years	Mechanical + technical

Abbreviations: MC, metal–ceramic; MZ, monolithic zirconia; Pro, prospective study; ReS, retrospective study; VZ, veneered zirconia.

### 3.3. Bias Assessments

Based on the total NOS scores, five studies were classified as having a low risk of bias, whereas two studies were considered to have a moderate risk of bias. No study was categorized as having a high risk of bias. Detailed NOS assessments are presented in Table 3.

**Table 3:** Bias assessment of selected articles based on the NOS tool.

Study, year	Selection			Comparability		Outcomes			Score
	A	B	C	D	E	F	G	H	
Pontevedra et al., 2024 [11]	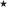	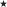	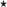	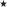	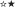	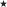	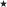	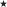	8
Zhang et al., 2024 [2]	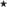	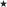	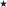	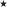	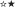	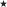	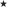	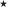	8
Solá‐Ruiz et al., 2022 [12]	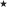	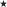	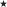	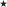	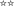	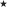	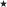	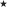	7
Pontevedra et al., 2022 [13]	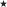	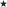	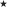	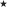	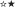	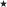	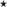	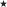	8
De Angelis et al., 2021 [14]	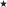	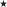	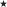	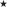	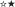	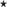	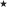	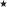	8
Diéguez‐Pereira et al., 2020 [15]	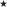	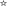	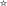	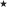	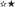	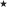	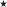	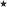	6
Wittneben et al., 2014 [16]	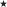	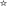	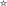	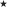	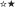	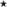	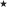	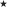	6

*Note:* A: representativeness of participants with exposure; B: selection criteria for participants without exposure; C: accuracy in determining exposure; D: indication that the outcome of interest was not present initially; E: demonstration that the outcome was absent at study commencement; F: evaluation of outcome measures; G: follow‐up period length; H: adequacy of follow‐up for all cohort members. Black stars (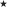) to signify that a study satisfactorily meets a specific criterion. White stars (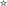) indicate that a criterion is not met.

### 3.4. Survival Rate

**Figure 2:**
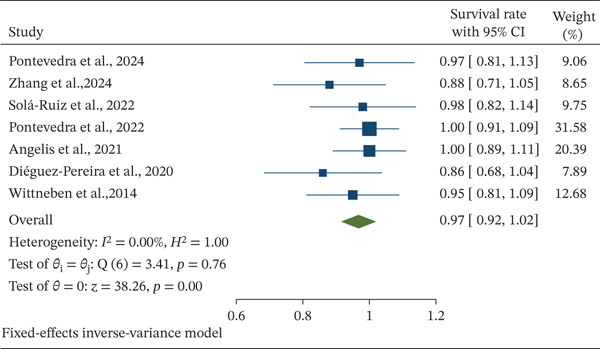
Forest plot showed the survival rate of screw‐retained ceramic‐veneered ISFDPs.

Using a fixed‐effects model with the inverse‐variance method, the survival rate of screw‐retained ceramic‐veneered ISFDPs was 97% (ES: 0.97; 95% CI: 0.92–1.09). No statistically significant heterogeneity was observed among the included studies (*I* ^2^ = 0.0*%*, *Q* = 3.41, *p* = 0.7565) (Figure 2).

### 3.5. Success Rate

**Figure 3:**
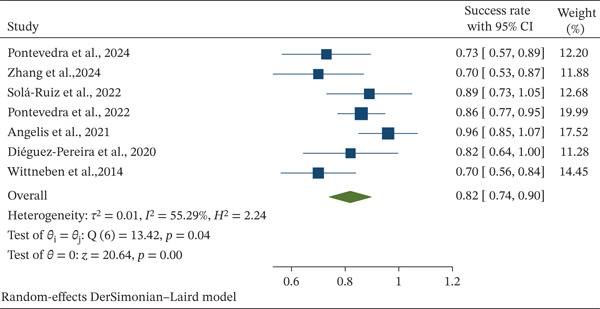
Forest plot showed the success rate.

Using a random‐effects model with the DerSimonian–Laird method, the success rate of screw‐retained ceramic‐veneered ISFDPs was 82% (ES: 0.82; 95% CI: 0.74–0.90). A moderate and statistically significant level of heterogeneity was observed among the included studies (*I* ^2^ = 55.3*%*, *τ* ^2^ = 0.0058, *Q* = 13.42, *p* = 0.04) (Figure 3).

### 3.6. Complication Rate

**Figure 4:**
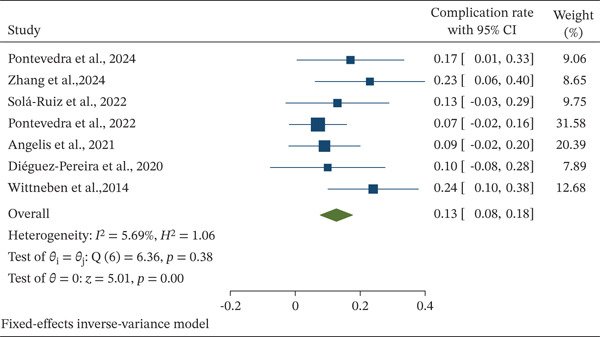
Forest plot showed the complication rate of screw‐retained ceramic‐veneered ISFDPs.

Using a fixed‐effects model with the inverse‐variance method, the complication rate of screw‐retained ceramic‐veneered ISFDPs was 13% (ES: 0.13; 95% CI: 0.08–0.18). Low heterogeneity was observed among the included studies (*I* ^2^ = 5.7*%*, *Q* = 6.36, *p* = 0.3839) (Figure 4).

Sensitivity analysis was performed by excluding studies with a moderate risk of bias [15, 16]. The pooled survival and complication rates remained stable, with no significant changes observed, indicating the robustness of the results.

## 4. Discussion

The present meta‐analysis demonstrated a high survival rate (96.8%) and a moderate success rate (81.9%) for screw‐retained ceramic‐veneered ISFDPs during long‐term follow‐up. The included studies reported survival outcomes at 3‐, 5‐, and 10‐year intervals, indicating that favorable survival and success rates were maintained over extended observation periods. The high survival rate observed in screw‐retained ceramic‐veneered ISFDPs may be attributed to improved passive fit, retrievability, and reduced risk of cement‐related biological complications, which are particularly relevant in long‐span implant prostheses. These findings are consistent with previous studies reporting comparable outcomes over shorter follow‐up durations, suggesting predictable long‐term performance of this prosthetic design [17, 18]. The pooled complication rate observed in the present analysis was 13%, which can be considered relatively low in comparison with other implant‐supported prosthetic reconstructions. However, this finding should be interpreted with caution from a clinical standpoint, as it may still represent a relevant long‐term maintenance burden in practice. The complication profile indicates that mechanical issues, particularly ceramic chipping and screw loosening, remain the primary limitations, suggesting that patient‐related factors such as bruxism and occlusal overload should be considered key determinants in treatment planning rather than implant survival alone. The most frequently reported technical complications associated with screw‐retained ceramic‐veneered ISFDPs included screw loosening, screw fracture, ceramic chipping, prosthesis fracture, proximal contact loss, and loss of implant access hole sealing material. Ceramic chipping in veneered zirconia frameworks is a well‐documented limitation of bilayered prosthetic designs, primarily related to differences in thermal expansion coefficient between the zirconia core and the veneering ceramic, as well as residual tensile stresses introduced during the cooling phase of fabrication. In addition, occlusal overload and parafunctional habits such as bruxism may further exacerbate veneer fracture risk over time. Compared with monolithic zirconia restorations, which eliminate the veneering layer and therefore reduce the risk of chipping, veneered systems generally demonstrate a higher incidence of technical complications despite their superior esthetic outcomes. A relevant distinction between veneered zirconia and monolithic zirconia restorations is the predictability of the fabrication workflow. Veneered zirconia prostheses involve manual layering techniques that are highly dependent on technician skill, which may introduce variability in the final restoration quality. In contrast, monolithic zirconia restorations are primarily fabricated through digital workflows, offering greater standardization, reproducibility, and consistency in production. This difference may be clinically relevant, particularly in the transition from provisional restorations to definitive prostheses, where consistency in material behavior and fabrication accuracy can influence the predictability of the final outcome. Nevertheless, these complications do not necessarily compromise overall implant survival, as most chipping events are minor and manageable through polishing or repair. However, recurrent or severe chipping may negatively affect prosthesis longevity and increase maintenance demands, thereby influencing the long‐term success rate rather than survival alone. Ceramic chipping was the most prevalent complication, a finding that aligns with previous reports on veneered zirconia restorations. In contrast, only a limited number of biological complications were reported across the included studies. Bruxism has been identified as an important risk factor that may negatively affect both implant and prosthetic survival rates by increasing mechanical overload and the incidence of technical complications. Several studies have demonstrated that severe bruxism is associated with a higher incidence of technical complications, particularly ceramic fractures and chipping [19, 20]. Moreover, it has been reported that ceramic chipping can reduce the survival of implant‐supported prostheses by up to 50% [21]. Consequently, patient‐related factors such as parafunctional habits should be carefully assessed during treatment planning.

Unlike previous meta‐analyses focusing on general ISFDPs or mixed retention systems, the present study specifically isolates screw‐retained ceramic‐veneered ISFDPs, providing more focused evidence for this prosthetic design. Previous systematic reviews, including Pozzi et al. and Pjetursson et al. [4, 7], have reported consistently high survival rates for ISFDPs across various prosthetic designs and retention modalities. However, these studies primarily pooled heterogeneous prosthetic configurations, including both screw‐ and cement‐retained systems, as well as different framework and veneering materials. In contrast, the present systematic review and meta‐analysis specifically focused on screw‐retained ceramic‐veneered ISFDPs, thereby providing more homogeneous evidence for this particular prosthetic design. This methodological focus reduces clinical and methodological heterogeneity related to retention type and material combination, allowing for more precise estimation of survival and complication outcomes in this subgroup. Previous systematic reviews have suggested that conventionally veneered zirconia restorations may not be recommended due to their higher susceptibility to fracture and chipping. However, comparative evidence indicates that both buccal‐veneered zirconia and monolithic zirconia ISFDPs can provide satisfactory clinical outcomes over 5‐ and 10‐year follow‐up periods [22]. These findings suggest that appropriate material selection and prosthetic design may effectively reduce complication rates while maintaining high survival outcomes, supporting their widespread clinical application.

A degree of clinical and methodological heterogeneity should be acknowledged when interpreting the pooled results of the present meta‐analysis. The included studies exhibited variability in prosthesis design, ranging from single‐unit to multiple‐unit fixed dental prostheses, as well as differences in material combinations, including monolithic zirconia, veneered zirconia, and metal–ceramic restorations. In addition, follow‐up durations varied considerably across studies. These factors may have contributed to observed statistical heterogeneity, particularly in outcome measures such as success rate. Therefore, while the pooled estimates provide an overall summary of available evidence, they should be interpreted with caution in the context of these clinical and methodological differences.

From a clinical perspective, the findings of this meta‐analysis support the use of screw‐retained ceramic‐veneered ISFDPs as a predictable and reliable treatment option for partially and completely edentulous patients. Clinicians should be aware that while long‐term survival is excellent, a proportion of prostheses may experience biological or technical complications over time. Therefore, careful case selection, meticulous prosthetic planning, and regular maintenance remain essential to optimize long‐term outcomes.

Several limitations of the present meta‐analysis should be acknowledged. First, moderate heterogeneity was observed in the success rate analysis (*I* ^2^ = 55.3*%*), indicating variability among the included studies. This heterogeneity may be attributed to differences in the definition of “success” across studies, as some investigations considered any biological or technical complication as a failure, whereas others allowed for minor, manageable events. Variations in follow‐up duration, prosthetic design, veneering techniques, and clinical protocols may have further contributed to this inconsistency. In contrast, no heterogeneity was detected in the survival rate analysis (*I* ^2^ = 0.0*%*), and low heterogeneity was observed for complication rates (*I* ^2^ = 5.7*%*), suggesting that these outcomes were consistently reported across studies. However, despite this consistency, the relatively small number of included studies may limit the generalizability of the pooled estimates. Another limitation was the incomplete reporting of patient‐related risk factors, such as bruxism, smoking status, systemic diseases, medication use, and history of periodontal disease. These variables may influence implant and prosthetic outcomes and should be investigated in future studies. Most of the included studies were retrospective in design, which may introduce selection bias and limit causal inference. Future well‐designed prospective studies and RCTs with standardized definitions of success and longer follow‐up periods are needed to reduce heterogeneity and improve the comparability of outcomes. A key limitation of the present meta‐analysis is that subgroup and sensitivity analyses were initially planned to further explore sources of heterogeneity. However, due to the limited number of included studies within specific subgroups and the inconsistent reporting of key variables (such as prosthesis design and complication definitions), meaningful subgroup stratification was not statistically feasible without compromising the interpretability of the findings. This should be considered when interpreting the pooled results. A further limitation relates to the variability in the definition of “success” across the included studies, which may have influenced the pooled success rate and limited its comparability. Therefore, the success rate should be interpreted with caution. The lack of PROSPERO registration was also another limitation, as it may affect transparency and reproducibility.

## 5. Conclusion

Screw‐retained ceramic‐veneered ISFDPs demonstrated high survival rates with a moderate success rate and relatively low complication rates. The main complications observed were related to technical issues, particularly ceramic chipping and fractures. The findings of the present study indicate an acceptable performance of these prostheses within the context of the available evidence. Randomized controlled trials with appropriate design, standardized definitions of success, and longer follow‐up periods are required to improve the quality of evidence and enhance the precision of future findings.

## Author Contributions

Concept: M.S. and J.H.G. Design: M.S., J.H.G., A.S., and M.R. Supervision: M.S., J.H.G., A.S., and M.R. Funding: M.S., J.H.G., A.S., and M.R. Materials: M.S., J.H.G., A.S., and M.R. Data collection and/or processing: M.S., J.H.G., A.S., and M.R. Data analysis and/or interpretation: M.S., J.H.G., A.S., and M.R. Literature search: M.S., J.H.G., A.S., and M.R. Writing: M.S. and J.H.G.

## Funding

No funding was received for this manuscript.

## Conflicts of Interest

The authors declare no conflicts of interest.

## Data Availability

The data that support the findings of this study are available upon request from the corresponding author. The data are not publicly available due to privacy or ethical restrictions.
